# Music for autism: a protocol for an international randomized crossover trial on music therapy for children with autism

**DOI:** 10.3389/fpsyt.2023.1256771

**Published:** 2023-10-02

**Authors:** Marianna Ruiz, Alexander Groessing, Alexandrina Guran, Asena U. Koçan, Nace Mikus, Urs M. Nater, Karlijn Kouwer, Maj-Britt Posserud, Maayan Salomon-Gimmon, Boryana Todorova, Isabella C. Wagner, Christian Gold, Giorgia Silani, Karsten Specht

**Affiliations:** ^1^Department of Health and Social Sciences, Norwegian Research Centre (NORCE), Bergen, Norway; ^2^Department of Biological and Medical Psychology, Faculty of Psychology, University of Bergen, Bergen, Norway; ^3^Department of Clinical and Health Psychology, Faculty of Psychology, University of Vienna, Vienna, Austria; ^4^Social, Cognitive and Affective Neuroscience Unit, Department of Cognition, Emotion, and Methods in Psychology, Faculty of Psychology, University of Vienna, Vienna, Austria; ^5^Vienna Cognitive Science Hub, University of Vienna, Vienna, Austria; ^6^School of Culture and Society, Interacting Minds Centre, Aarhus University, Aarhus, Denmark; ^7^Division of Psychiatry, Haukeland University Hospital, Bergen, Norway; ^8^Department of Clinical Medicine, Faculty of Medicine, University of Bergen, Bergen, Norway; ^9^The School of Creative Arts Therapies, Faculty of Social Welfare and Health Sciences, University of Haifa, Haifa, Israel; ^10^Department of Cognition, Emotion, and Methods in Psychology, Faculty of Psychology, University of Vienna, Vienna, Austria; ^11^Centre for Microbiology and Environmental Systems Science, University of Vienna, Vienna, Austria; ^12^Department of Radiology, Mohn Medical Imaging and Visualization Centre, Haukeland University Hospital, Bergen, Norway; ^13^Department of Education, Faculty of Humanities, Social Sciences and Education, UiT-The Arctic University of Norway, Tromsø, Norway

**Keywords:** autism spectrum, music therapy, social communication, brain connectivity, protocol, randomized controlled trial

## Abstract

The notion of a connection between autism and music is as old as the first reported cases of autism, and music has been used as a therapeutic tool for many decades. Music therapy holds promise as an intervention for individuals with autism, harnessing their strengths in music processing to enhance communication and expression. While previous randomized controlled trials have demonstrated positive outcomes in terms of global improvement and quality of life, their reliance on psychological outcomes restricts our understanding of underlying mechanisms. This paper introduces the protocol for the Music for Autism study, a randomized crossover trial designed to investigate the effects of a 12-week music therapy intervention on a range of psychometric, neuroimaging, and biological outcomes in school-aged children with autism. The protocol builds upon previous research and aims to both replicate and expand upon findings that demonstrated improvements in social communication and functional brain connectivity following a music intervention. The primary objective of this trial is to determine whether music therapy leads to improvements in social communication and functional brain connectivity as compared to play-based therapy. In addition, secondary aims include exploring various relevant psychometric, neuroimaging, and biological outcomes. To achieve these objectives, we will enroll 80 participants aged 6–12 years in this international, assessor-blinded, crossover randomized controlled trial. Each participant will be randomly assigned to receive either music therapy or play-based therapy for a period of 12 weeks, followed by a 12-week washout period, after which they will receive the alternate intervention. Assessments will be conducted four times, before and after each intervention period. The protocol of the Music for Autism trial provides a comprehensive framework for studying the effects of music therapy on a range of multidimensional outcomes in children with autism. The findings from this trial have the potential to contribute to the development of evidence-based interventions that leverage strengths in music processing to address the complex challenges faced by individuals with autism.

**Clinical Trial Registration**: Clinicaltrials.gov identifier NCT04936048.

## 1. Introduction

Autism spectrum disorder (ASD) is a highly heterogeneous, lifelong neurodevelopmental condition that is diagnosed based on behavioral criteria. It is characterized by challenges in social communication and interaction, as well as restricted and repetitive patterns of interests and behavior that significantly impact daily life (1). Autism symptoms vary extensively between individuals and can manifest differently within the lifespan of a single individual (2). The presence of co-occurring psychiatric and medical conditions further contributes to the complexity and heterogeneity of autism, making it challenging to identify effective treatment approaches for individuals (3, 4).

Currently, early and intensive behavioral interventions are recommended as the primary treatment to target core ASD symptoms (5, 6). However, these studies are limited by methodological constraints (7, 8), and the cost and demanding nature of implementing early intensive behavioral interventions pose barriers to their accessibility (9). Consequently, there are limited evidence-based treatment options available, and the sole focus on reducing core behavioral symptoms may not adequately address the multifaceted mental health needs of individuals with autism (10).

Music therapy (MT) has been used for several decades to enable communication and expression in individuals with autism (11). In contrast to difficulties in social processing, music processing has been identified as a strength and an interest for many individuals with autism since the initial descriptions of autism (12) and remains a topic of ongoing research. Studies focusing on auditory processing indicate that individuals with autism exhibit a preference for musical over verbal stimuli (13, 14). Furthermore, parental reports suggest children with autism often choose to engage in musical activities and that music elicits an enduring emotional response in them (15–17).

Beyond showing an interest in music, musical abilities have also been considered a relative strength within their global profile (18, 19). Several studies have reported better performance in individuals with autism versus controls on tasks related to pitch processing (for review, see (15)), enhanced melodic memory (17, 20, 21), and intact rhythm synchronization (22). Notably, while individuals with autism may encounter challenges in processing emotions from social stimuli (23), research suggests that they do not experience difficulties in understanding emotions expressed through music, known as musical emotions (24–26). Therefore, music processing represents a domain in which some individuals with autism exhibit strengths and a notable level of interest. Moreover, music serves as an easily accessible and widely available stimulus that effectively engages and emotionally rewards children with autism (27, 28). Thus, music therapy provides an opportunity to leverage strengths in music processing to address the challenge of developing social communication skills.

Systematic and robust scientific investigation of the effects of music therapy (MT) in this population began only recently (29). Drawing from 26 randomized controlled trials (RCTs) conducted since the 1990s, MT has demonstrated positive outcomes in terms of global improvement, symptom severity, and improved quality of life (11). However, findings from a large-scale RCT indicated that MT may not result in significant changes in core symptoms as assessed by diagnostic tools (30). It is important to note that the majority of RCTs conducted on MT thus far have primarily relied on observations of behaviors that are clinically relevant as outcome measures (9). To gain a deeper understanding of the underlying mechanisms of MT, it is crucial to establish connections between behavioral outcomes and neuroscientific measures, such as resting-state functional connectivity (RSFC) obtained from functional magnetic resonance imaging (fMRI) (31).

Resting-state functional magnetic resonance imaging (rsfMRI) is a valuable tool for studying functional connectivity (FC) in clinical and pediatric populations. Unlike task-based fMRI, which measures responses to specific tasks, rsfMRI examines the organization of intrinsic functional networks that are stable across different behavioral states (32, 33). By scanning subjects in a wakeful state without engaging in specific tasks, rsfMRI removes some cognitive demands and enables the examination of clinical populations more effectively. It also allows for scanning subjects at multiple time points, eliminating repetition effects associated with task-based paradigms (34).

RSFC studies have reported atypical connectivity within and between functional brain networks in individuals with ASD (35–37). Initial research suggested general cortical hypoconnectivity as a possible explanation for clinical symptoms in ASD (38–40). However, divergent findings have emerged, including hypoconnectivity, hyperconnectivity, and a combination of both in individuals with ASD compared to controls [for review, see (35)]. Age plays a crucial role in understanding FC in ASD, as the brain undergoes significant developmental changes that might help explain divergent findings (36). From a developmental perspective, emerging evidence suggests both short- and long- range hyperconnectivity in school-aged children with autism relative to typically developing children (36, 41–43). Although the precise characteristics of brain connectivity in ASD remain unclear, accumulating evidence suggests the involvement of both hypo- and hyperconnectivity, with children showing differences in overall FC patterns to those seen in adolescents and adults.

To date, only one randomized controlled trial (RCT), namely Sharda et al. (29), has integrated both behavioral outcomes and functional brain connectivity to evaluate the effects of music interventions on school-aged children with autism. Sharda et al. selected six target seed areas (bilateral Heschl’s gyrus, HG; inferior frontal gyrus, IFG; temporal pole, TP) based on their relevance to language and communication, as well as a prior study on perception of sung versus spoken words (44). The findings from the seed-based connectivity analysis indicated an increase in connectivity between auditory seeds (left and right HG) and striatal and motor regions, as well as a decrease in connectivity between auditory seeds (left HG and right TP) and visual areas following 8–12 weeks of music intervention compared to a non-music play-based intervention. Importantly, these changes in connectivity were correlated with improvements in social communication scores as rated by parents, providing initial evidence that music interventions can improve both social communication and functional brain connectivity in children with autism (29). However, there is a need for replication and expansion of these findings, and this is the aim of the Music for Autism (M4A) trial.

The M4A trial will investigate various additional outcomes and potential mechanisms than those studied by Sharda et al. These include exploring the impact of MT on chronic stress, an often overlooked issue among individuals with ASD (10) that has been shown to impede learning (45) and that can be modulated through musical interventions (46). The study will also examine potential structural brain changes, which are known to be influenced by psychological and biological therapies (47) as well as musical training (48). Furthermore, the research will delve into the concept of predictive processing, exploring potential links between prediction and pleasure derived from music (49), and it will examine children’s participation in the community, which is known to be limited by environmental barriers (50). Lastly, brain structure and function are closely tied to the gut microbiome (i.e., the microorganisms that inhabit the gastrointestinal tract) (51). The study aims to both examine the gut-brain axis in children with ASD (52, 53) and to explore whether music therapy intervention could serve as a potential modulator of effects.

### 1.1. Aims

The primary aim of the Music for Autism (M4A) trial is to investigate whether a 12-week music therapy intervention, compared to play-based therapy, can lead to improvements in social communication and functional brain connectivity in school-aged children with autism. Secondary aims include examining the effects of MT on a range of psychometric, neuroimaging, and biological outcomes in children with autism.

We hypothesize that a 12-week music therapy (MT) intervention, compared to play-based therapy (PT), will lead to the following outcomes in children with autism aged 6–12:

1. Psychometric outcomes:

- Significant improvements in *social communication* (primary).
- Increased *participation* in home, school, and community life.
- Improvements in *family quality of life*.
- Increased *adaptive behaviors*.
- Improvements in *receptive vocabulary* skills.
- Improvements in *social functioning*.
- Improved *prediction* accuracy.

2. Neuroimaging outcomes:

- Changes in *resting-state functional connectivity* (primary), characterized by:
  - Increased coupling between auditory and both striatal and motor regions.
  - Decreased coupling between auditory and visual regions.
- Increased whole-brain *grey and white matter volume.*

3. Biological outcomes:

- Reduced hair cortisol concentration, indicating a decrease in *chronic stress*.
- Alterations in *gut microbiome* composition and an association between changes in gut microbiome composition and changes in neuroimaging and psychometric outcomes.

Furthermore, we anticipate that the observed changes in functional connectivity will correlate with the primary outcome of improved social communication skills.

## 2. Methods and analysis

### 2.1. Study design

**Figure 1:**
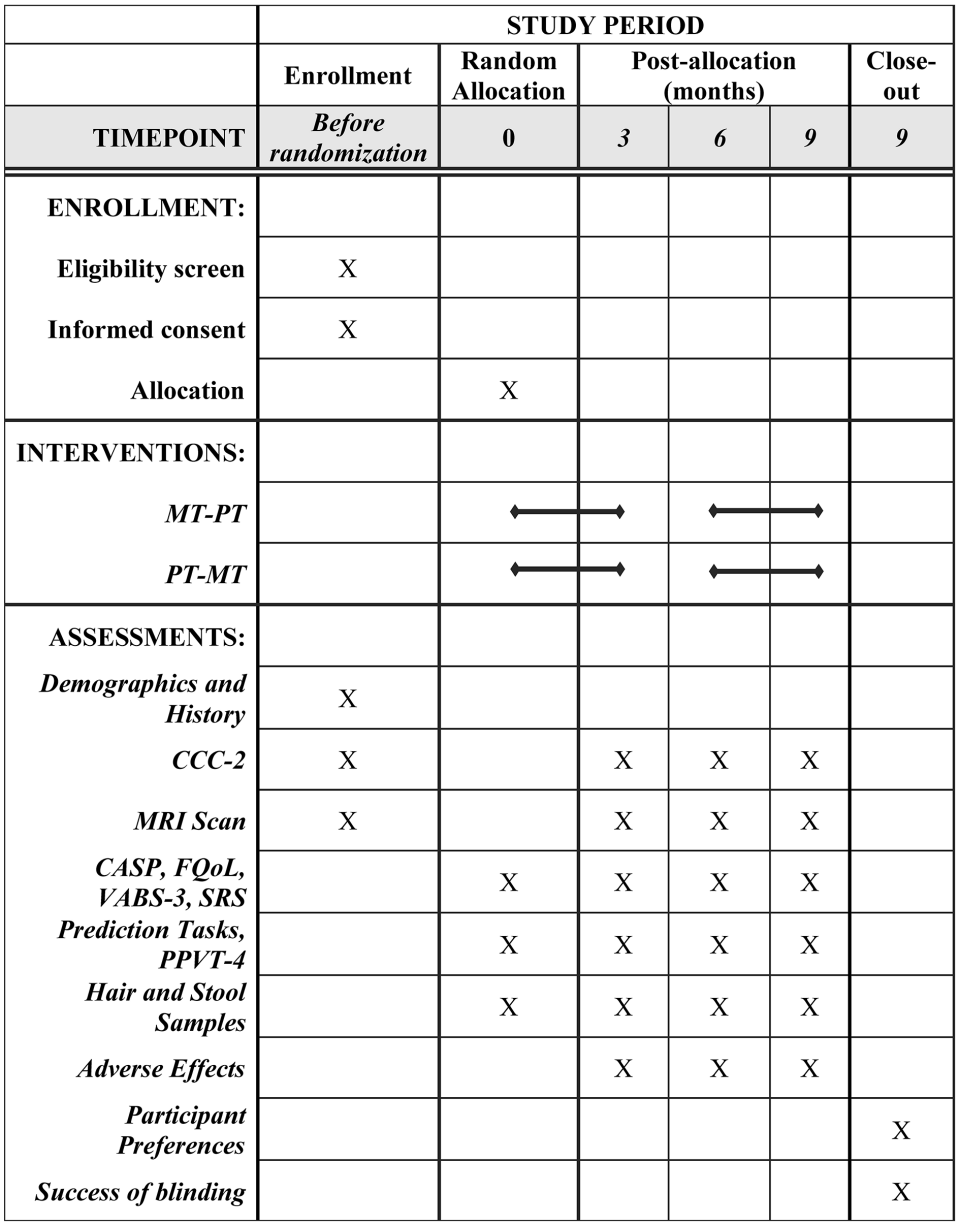
Schedule of enrollment, interventions, and assessments. MT, Music therapy; PT, Play therapy; MRI, Magnetic Resonance Imaging; CCC-2, Children’s Communication Checklist 2nd Edition; CASP, Child and Adolescent Scale of Participation; FQoL, Beach Centre Family Quality of Life Scale; VABS-3, Vineland Adaptive Behavior Scales 3rd Edition; SRS, Social Responsiveness Scale; PPVT-4, Peabody Picture Vocabulary Exchange Test 4th Edition.

M4A is an international, assessor-blinded crossover RCT comparing music therapy (MT) to a structurally matched play-based therapy (PT) intervention, combining psychometric, neuroimaging and biological outcome measures (Figure 1).

**Figure 2:**
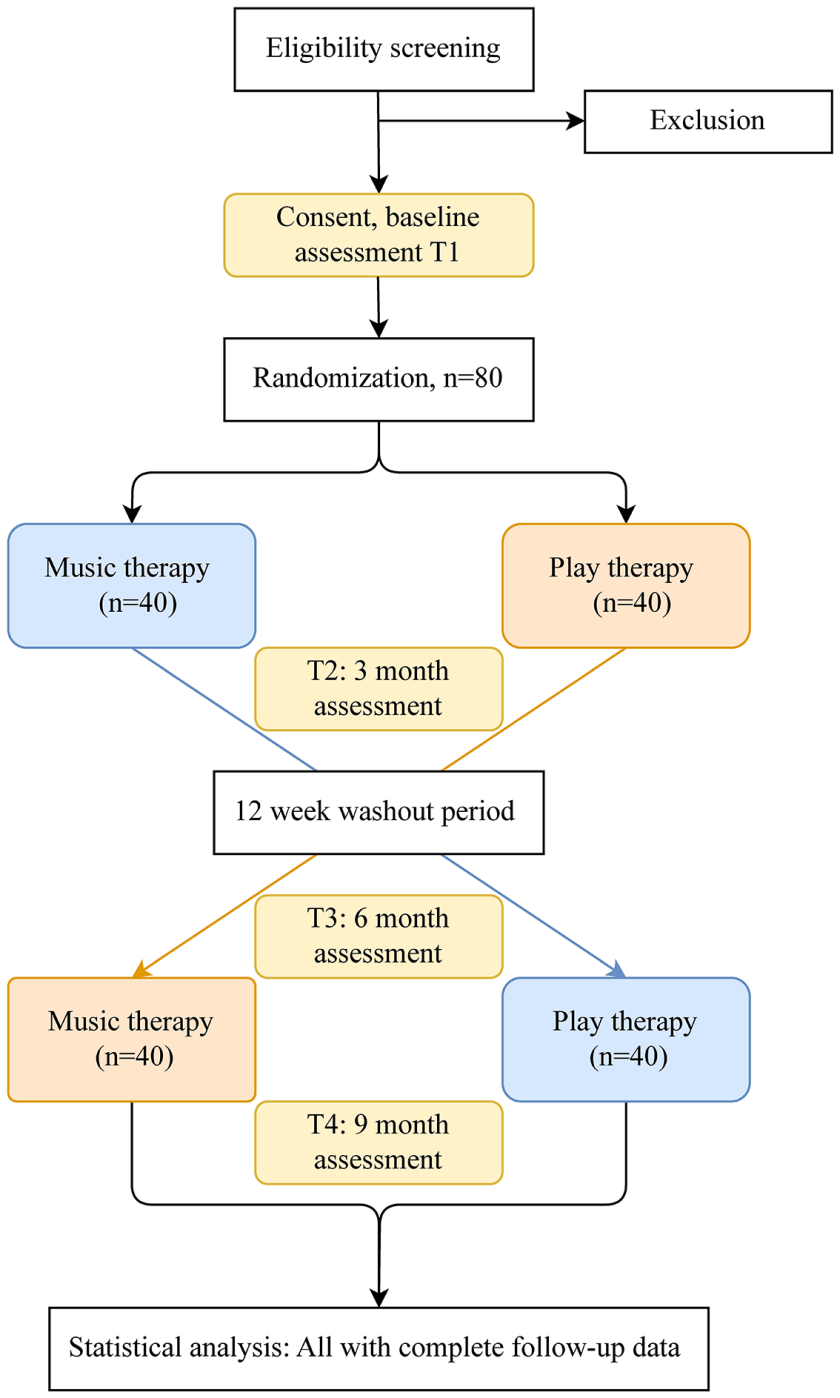
Flow of participants through the study: illustration of the study design. T1, Baseline assessment/pre-intervention at 0 months; T2, Post-intervention 1 at 3 months; T3, Pre-intervention 2 at 6 months; T4, Post-intervention 2 at 9 months.

Following consent and baseline assessment, 80 participants will be randomized to a sequence of interventions (MT-PT or PT-MT). The first intervention will take place over 12 weeks, followed by a washout period of 12 weeks, and then by 12 weeks of the second intervention. Assessments will be conducted at four time points, before and after each intervention period (Figures 1, 2). Changes to the protocol since the initial version are listed in Supplementary Table S1.

### 2.2. Participants

Inclusion criteria are being 6–12 years old and having an ASD diagnosis from a licensed health professional with an ICD code (54), ideally supported with scores of standardized tools [ADOS (55), ADI-R (56)] and assessment of intelligence quotient (IQ) or level of ability. Exclusion criteria include individual MT during the last 6 months; generalized epileptic seizures during the last 12 months; more than 12 months of cumulative music training; or conditions precluding fMRI scanning (such as metallic or electronic implants, claustrophobia, hyperacusis, or persistent problems in complying with scanning procedures).

#### 2.2.1. Recruitment

Participants will be recruited in two cities (Bergen, Norway; Vienna, Austria) by advertising through social media and reaching out to relevant groups such as parent associations, schools, clinicians, and special educators.

#### 2.2.2. Enrollment and randomization

When possible, enrollment will be scheduled so that interventions will take place during the school year and washout periods during school holidays. We expect to randomize around 27 participants each half year in both sites combined. Computer-generated randomization lists, with randomly varying block sizes of 4–6, separate for each country, will be used to ensure both unpredictability and balance. The lists will be generated by a researcher who does not have contact with participants and are stored centrally, concealed from clinical investigators, until a decision on inclusion is made. Once a participant is enrolled, the randomization result will be conveyed to clinical investigators through an online system (Research Electronic Data Capture (REDCap) on Norwegian Research Centre (NORCE) servers).

### 2.3. Interventions

Locations (a dedicated room at each site) and materials (matched to those in Sharda et al. as closely as possible) will be standardized to ensure consistent implementation in Bergen and Vienna. Both interventions will consist of 12 weekly one-on-one sessions, 45 min each, conducted in the same setting by a licensed music therapist. In sum, both MT and PT use a developmentally oriented approach with the overall aim of improving quality of life (57) and targeting creating a shared experience, building meaningful relationships, and fostering self-expression. A varied set of activities, combining therapist- and child-led interactions, target common goals including sensory processing, emotional development, and social communication.

Children can choose 4 activities per session using pictures of the activities and a visual schedule to facilitate communication and provide structure to the sessions (58). MT uses rhythmic cues, musical instruments (e.g., piano, djembe, xylophone, ukulele), songs, and stories accompanied by songs. PT is designed as a play-based active comparison condition to control for factors such as support, therapist attention, positive expectancies, and emotional engagement. It uses verbal interaction, toys (e.g., building bricks, finger puppets, plasteline, puzzles), and stories without a musical component.

Treatment fidelity (59, 60) will be monitored and ensured via therapists’ reports, supervision meetings, and video recordings of sessions. For every child, three videos from each intervention arm (first, middle, and last session) will be assessed by two independent raters. The fidelity checklist covers dimensions such as dose (e.g., number of sessions); structure (e.g., number and type of activities); content (e.g., use of musical or verbal reinforcements); and processes (e.g., therapist’s attunement, therapist’s responsiveness). In addition, child engagement and interaction levels will be investigated. Differentiation between MT and PT will be analyzed by comparing the amount of musical and verbal reinforcement used.

### 2.4. Outcome measures

The primary outcomes are social communication and functional brain connectivity. Secondary outcomes include a range of psychometric, neuroimaging and biological outcomes. For an overview of all outcome measures see Table 1.

**Table 1:** Overview of outcomes and measures.

Outcome	Measure (source)
*Psychometric*	
Social communication*	CCC-2 (t)
Participation	CASP (p)
Family quality of life	FQoL (p)
Adaptive behavior	VABS-3 (p)
Social functioning	SRS (p)
Receptive vocabulary	PPVT-4 (c)
Predictive processing	Prediction tasks (c)
*Neuroimaging*	
Brain connectivity frontotemporal networks*	RSFC 6 seeds: HG, TP, IFG (c)
Structural brain changes	Whole-brain VBM (c)
*Biological*	
Chronic stress	Hair cortisol concentration (c)
Gut microbiome	16S rRNA gene amplicon sequencing (c)

*Primary outcomes.

c, child; p, parent; t, teacher.

CCC-2, Children’s Communication Checklist 2nd Edition; CASP, Child and Adolescent Scale of Participation; FQoL, Beach Centre Family Quality of Life Scale; VABS-3, Vineland Adaptive Behavior Scales 3rd Edition; SRS, Social Responsiveness Scale; PPVT-4, Peabody Picture Vocabulary Exchange Test 4th Edition; RSFC, Resting state functional connectivity; HG, Heschel’s gyri; TP, Temporal poles; IFG, Inferior frontal gyri; VBM, Voxel-Based Morphometry.

#### 2.4.1. Psychometric outcomes

Psychometric outcomes include: (1) social communication (primary); (2) participation; (3) family quality of life; (4) receptive vocabulary; (5) social functioning; (6) adaptive behavior; and (7) predictive processing. Participation and family quality of life are the two main secondary psychometric outcomes because of their particular importance to participants. All outcomes except social communication, receptive vocabulary, and predictive processing are parent-rated.

(1) *Social communication* as assessed by a blinded assessor using the Children’s Communication Checklist 2nd Edition (CCC-2) (61, 62) will be the primary psychometric outcome. The CCC-2 measures aspects of pragmatic communication with 70 items across 10 subdomains (speech, syntax, semantic, coherence, inappropriate initiation, stereotyped language, use of context, nonverbal communication, social relations, and interests) and has shown high interrater reliability and internal consistency (40). The General Communication Composite (GCC) standard score is standardized to *M* = 100 (SD = 15), with higher scores indicating better social-communication skills (reversed from raw scores). Whereas Sharda et al. relied on parent reports for this outcome, where attempted blinding was unsuccessful (29), M4A relies on a special educator/teacher who knows the child well. Success of blinding will be verified at the last follow-up by asking the assessor about incidental unblinding.
(2) *Participation* is measured using the Child and Adolescent Scale of Participation (CASP), a caregiver-report scale consisting of 20 items across four subdomains (home participation, community participation, school participation, home and community living activities) (63). The scale has high test–retest reliability and internal consistency. The 20 items are rated on a four-point scale from 1 (unable to participate) to 4 (age-expected/full participation). Parents can indicate if any of the items does not apply to them. The sum of all applicable items is then divided by the maximum possible sum score (as given by the number of applicable items) and multiplied by 100 to produce a score with a possible range from 0 and 100. Higher scores indicate more participation.
(3) *Family quality of life* is measured using the Beach Center Family Quality of Life Scale (FQoL) (64), a 25-item scale to measure families’ perceived satisfaction in terms of quality of family life (including family interaction, parenting, emotional well-being, physical/material well-being, and disability-related support). Parents rate each item on a 5-point scale from 1 (very dissatisfied) to 5 (very satisfied) for a total sum score from 25 and 125, with higher scores indicating better quality of life.
(4) *Receptive vocabulary* is measured with the Peabody Picture Vocabulary Test-4th edition (PPVT-4) (65, 66), conducted by a qualified health professional. The assessment with up to 228 items (each with a spoken word +4 pictures to choose from) produces raw scores which are then converted to standard scores using a conversion table. We will use German norms in Austria, and American norms (in the absence of Norwegian norms) in Norway. The German manual deviates from the American manual in suggesting T-scores (50 ± 10) instead of standard scores (100 ± 15); however, we will convert all T-scores to standard scores [i.e., (x–50)*1.5 + 100 for the Austrian data] to ensure comparability. Handling of extreme values is also different: While the American norms used in Sharda et al. and in the Norwegian M4A sample allow for a wide range of scores, Austrian standard scores are cut off at ±2.3 SD according to the German manual (65). Higher scores indicate better receptive vocabulary.
(5) *Social functioning* will be assessed through the Social Responsiveness Scale (SRS-2) (67). Parents are asked to rate 65 items associated with behaviors observed in autism (in 5 subdomains: social awareness, social communication, social motivation, social cognition, and restricted and repetitive behaviors), each from 1 (not true) to 4 (almost always true). Total raw scores are converted into T-scores (50 ± 10) through a conversion table. Higher scores on the SRS indicate greater social difficulties, while lower scores indicate better social functioning. The instructions normally ask respondents to consider the past 6 months; we adjusted this to the past 6 weeks to match our intervention periods.
(6) *Adaptive behavior* will be assessed using the Vineland Adaptive Behavior Scales, 3rd Edition (Vineland-3) (68). It is a comprehensive parent-rated assessment tool used to measure adaptive behavior and to support the diagnosis of intellectual and developmental disabilities, autism, and developmental delays. Vineland maladaptive behavior as well as gross and fine motor skills are reported as v-scale scores (standardized 15 ± 3 in the general population). In addition to the domains used by Sharda et al., M4A also uses the Adaptive Behavior Composite (ABC) standard score (100 ± 15), from the domains of communication, daily living skills, and socialization, as a measure of overall adaptive functioning level. Higher adaptive behaviors and gross and fine motor skills scores, and lower maladaptive behavior scores, represent better outcomes.
(7) *Predictive processing* will be assessed through two behavioral prediction tasks. Children will be asked to complete two tasks on a computer: a music prediction task, and an action prediction task to evaluate (a) their ability to learn stimulus-outcome associations with both music stimuli and the actions of others; and (b) the degree of pleasure they derive from successful predictions. Both tasks combined last around 40 min.

In the music prediction task, children will be presented with different music pieces that will end either in an expected, consonant way, or with an unexpected, dissonant sequence of tones. Each type of music is associated with the portrait of one composer (in a comic drawing style) in the training phase. In the experimental phase, children are asked, after listening to the music, how much they like the music and which of the two composers was playing it.

In the action prediction task, children are presented with several videos displaying the unfolding of an action: taking or giving an apple or a glass of water. Each action is predicted by environmental cues (e.g., red or violet plate for the apple, white or blue tablecloth for the glass). During the task children are asked to guess from an initial static picture whether the action is taking or giving (by taking into consideration the changing cue), how sure they are of their choice and, after seeing the video, how much they liked it.

#### 2.4.2. Neuroimaging outcomes

*Brain connectivity* of fronto-temporal brain networks will be derived from resting-state fMRI. Six seed areas will be used as the primary neuroscientific outcome. Seeds are anatomically defined regions of interest (ROIs) in Montreal Neurological Institute (MNI) space for the *left and right Heschel’s Gyri, Inferior Frontal Gyri, and Temporal Poles*. These seeds form part of fronto-temporal brain networks involved in language and communication that may be altered in ASD and modified by MT (29).

*Structural brain changes,* grey and white matter volume, will be assessed using whole-brain voxel-based morphometry (VBM), derived from the structural T1-weighted image acquired at the beginning of each scanning procedure.

#### 2.4.3. Biological outcomes

*Chronic stress* will be measured through hair cortisol concentration in the scalp-nearest 3 cm segment, reflecting cumulative cortisol secretion over the past 3 months (69). Chronic stress was suggested by M4A user representatives during the development of the initial protocol and is relevant to MT (46) and ASD (10). For determination of hair cortisol concentration, the first scalp-near 3 cm segment will be used which is thought to reflect the cumulative cortisol secretion of the past 3 months (70). Hair wash and cortisol extraction procedures are based on a laboratory protocol by Stalder et al. (69), with minor modifications (71). For cortisol determination, a commercially available cortisol luminescence immunoassay will be used (LIA; IBL International, a Tecan Group company, Hamburg, Germany). Inter- and intra-assay coefficients of variation will be computed.

*Gut microbiome data* will be collected from stool samples to investigate the relationship between the profile of gut microbial composition in children with ASD and brain structure and function and if music therapy influences the gut microbiome composition. The “gut-brain-axis” is thought to provide a bidirectional pathway of communication between the gut microbiome, brain function, and behavior, routed via the vagus nerve and other mechanisms (72–74). ASD is often associated with gastrointestinal problems and gut microbiota dysfunction (75). How the gut-brain axis affects cognitive function is unclear; an informed cognitive neuroscience perspective on this matter is so far missing. To account for factors potentially affecting gut microbial composition, we will also ask whether participants experienced intestinal illness and/or have taken antibiotics in the last 3 months, the individual’s body-mass index, dietary habits, as well as the frequency and timing of bowel movements. The microbial community composition in collected stool samples will be determined by 16S rRNA gene amplicon sequencing (76). The resulting data will be taxonomically classified and submitted to analyses using common bioinformatics pipelines implemented in R (77).

#### 2.4.4. Additional measures

Demographics (age, gender, and socioeconomic status) and developmental history will be collected at baseline.

*Adverse effects* of MT are rare (11, 30), but will be collected throughout the study. After each intervention, parents will be asked if any serious or non-serious adverse events occurred since the previous assessment, irrespective of their connection to the study procedures. Parents will also be requested to provide a detailed description of any adverse events encountered.

*Participant preferences* will be recorded by asking parents at the last follow-up to ask their children which intervention they preferred, to what degree, and why. The ability to directly compare preferences is unique to crossover trials and may yield important additional information (78, 79).

### 2.5. Scanning parameters

Both sites will use 3 T scanners (Bergen: GE Signa HDx, Vienna: Magnetom Skyra by Siemens Medical), equipped with a 32-channel head coil and a high-performance gradient system for fast, high-resolution whole-brain multiband echoplanar imaging. Resting-state EPI images will be obtained in 54 slices with voxel dimensions of 2.39 × 2.39 × 2.4 mm, covering the whole brain (TR = 800 ms, TE = 3.7 ms, matrix size, 88 × 88; field of view [FOV] 210 mm; flip angle 52°). Seven hundred and fifty volumes will be obtained in 10 min; additionally, participants will complete a high resolution sagittal T1-weighted structural scan (voxel dimensions: 1.2 × 1.05 × 1.05 mm, reduction factor of 2) with a duration of 6 min and 45 s. Acquisition protocols will be harmonized across sites and aligned with the ENIGMA ASD working group (e.g., duration of resting state ≥8 min).

### 2.6. Study procedure

Assessments at the four planned time points will be conducted during two in-person visits and through questionnaires administered electronically to parents and teachers. Assessments will include demographic data and developmental history (only at baseline), psychometric, neuroimaging, and biological assessments (Table 1). Assessments will be conducted by adequately qualified staff, separate from intervention providers.

During the first visit, children will complete a brain scan and parents will receive a stool sampling kit to be delivered back to researchers on the second visit. The sampling kit consists of a sterile tube, a feces catcher to prevent stool from coming in contact with water or detergents, one-way latex gloves, a Styrofoam box and thermal packs for transport. Stool samples will be collected by participants (with help of the parents if needed), using the materials provided to them by the research team. The sampling kit is specially designed for stool sampling, is widely employed, and is easy to use.

The brain scan procedure requires children to be as still as possible for approximately 16 min inside the MRI scanner. To facilitate the procedure, children will be allowed to watch movies during the scan – an entertaining movie (Tom and Jerry or a preferred movie) during the preparatory scans, structural T1-weighted scan, and breaks between scans, and a specifically designed video (80) during the resting state sequence of the scan that lasts 8 min. To help families prepare, we will provide parents with a movie showing the scanning environment and procedures, a recording of the sounds the scanner makes, and an individualized visual schedule showing the steps to follow on the day of the visit (Supplementary Figure S1).

On the day of the scan, researchers will maintain a calm and patient attitude to facilitate the experience and collaborate with radiographers about any specific details concerning the child. After the visit, children will receive a toy set of an MRI scanner which they can use to role-play visiting the scanner. When needed, we will also offer the option of meeting at the scanning facility prior to the baseline scan to familiarize children with the environment and the researchers.

During the second visit, children will complete a vocabulary assessment and two behavioral prediction tasks. Hair samples will be collected during this visit as well by cutting several thin hair strands as close as possible to the scalp from the posterior vertex region of the head. If a child does not want their hair to be cut, researchers will not collect the hair sample.

### 2.7. Statistical analysis

#### 2.7.1. Power calculation

**Figure 3:**
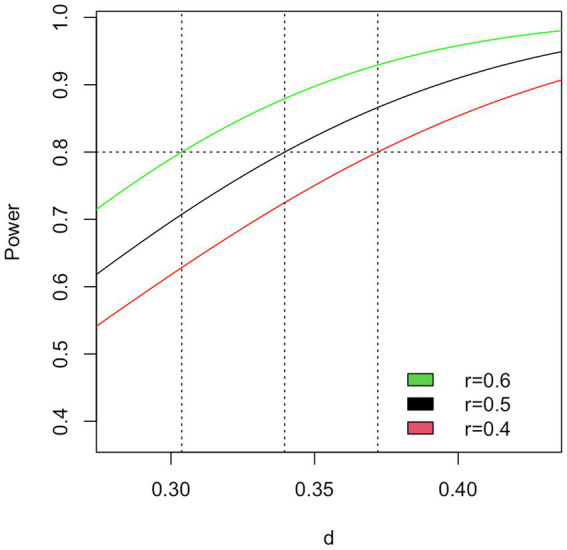
Power calculation for the primary outcome.

We designed M4A to be powered for an effect size of *d* = 0.34. Sharda et al. reported a mean difference of 4.84 and an effect size of *d* = 0.34 (SD not reported but calculated as 4.84/0.34 ≈ 14.2) on the CCC-2. Our previous Cochrane review showed similar effect sizes (81). We further assumed outcomes for MT and PT to be correlated with *r* = 0.50. With a two-sided significance level of 5%, a sample of *n* = 70 will be required for 80% power; to accommodate for attrition, we plan to recruit 80 participants. Similar power can be achieved for a range of reasonable effect sizes and correlations (Figure 3). The same power can be reached with varying combinations of effect sizes, correlations, and numbers of participants with complete data; in contrast, a parallel design would require almost 300 participants (Supplementary Table S2).

#### 2.7.2. Data analysis plan

Statistical analysis of all outcomes will be on an intention-to-treat (ITT) basis. In contrast to Sharda et al. who used a parallel design, special considerations apply to crossover trials. All continuous outcomes (whether psychometric, neuroimaging, or biological) will, following a graphical examination of normality, compare the post-test difference between interventions within each participant, adjusted by the difference in pre-tests within each participant in an ANCOVA model. This approach is optimal for crossover trials with baseline measurements, both in terms of making best use of baseline measures to improve precision, and in avoiding bias from randomly occurring baseline imbalance (82). The model can be written as follows:


$$\left(Y_{Ti}-Y_{\mathrm{Ci}}\right)=\beta_{T}+\gamma \left(X_{Ti}-X_{\mathrm{Ci}}\right)$$

for participant *i*, where *Y* is the post-test, *X* is the pre-test, *T* is the treatment and *C* is the control condition; the intercept β_T_ then represents the treatment effect and *γ* represents the influence of the baseline measurement (82). Sensitivity analyses will include age and functioning level as additional covariates. A further sensitivity analysis will include therapists as random effects.

The intention-to-treat principle will be followed as applicable in a crossover trial (79). Participants will be analyzed in the group to which they were randomized, regardless of whether they received the full allocated intervention. However, participants can only be analyzed if they have data for all time points for the respective outcome. This leads to two possible analysis strategies: (a) to include only participants with valid data for both intervention periods (complete case analysis); or (b) to impute values for missing values (either through multiple imputation based on known variables; or, as a simpler approach but with less desirable statistical properties, baseline values of the same outcome, known as last observation carried forward, LOCF). Sharda et al. used LOCF (personal communication, Megha Sharda, 4 April 2022). We aim to use complete case analysis as the main strategy unless the rate of missing data is high. If a complete case analysis results in a high proportion of missing cases (e.g., > 10%), we will consider an imputation strategy.

#### 2.7.3. Neuroimaging data analysis

Resting-state fMRI data will be preprocessed prior to statistical analysis. Preprocessing steps will include realignment with correction of susceptibility distortion interactions, slice timing correction, outlier detection, direct segmentation, normalization to MNI, and smoothing. We will examine the quality of fMRI scans using visual inspection and framewise displacement (FD) (83), an indicator of head motion, which is a common problem especially in children and clinical populations (84). Volumes exceeding a certain FD threshold [0.5 mm (83) or other values (85)] can be down weighted or scrubbed to obtain usable data (83); longer passages of high movement can be removed.

To examine resting-state functional connectivity (RSFC), the timeseries for each of the six seeds selected will be regressed against all other voxels timeseries in the brain to generate individual participant-level connectivity maps, using whole-brain general linear models at baseline and after the interventions. These first-level maps will then be entered into the second-level analyses. For comparison after the intervention, we will use ANCOVA with post-intervention RSFC as a dependent variable, and intervention, baseline RSFC, age, and IQ as covariates. Z-scores of parameter estimates will be used to measure connectivity strength. Results will be reported with a 5% significance level adjusted for multiplicity by familywise error rate.

To examine the relation between RSFC and behavior change, Z-statistics for each participant from the post-intervention RSFC maps will be used in a linear regression model to evaluate correlation between RSFC and behavior change. All tests will use a two-sided 5% significance level. The two main secondary psychometric outcomes, participation, and quality of life will be Bonferroni-corrected; the remaining secondary outcomes will be exploratory.

To examine structural brain changes images will be segmented into grey- and white-matter probability maps, and a voxel-wise whole-brain statistical analysis will be applied to detect any changes in grey and white matter volume. Preprocessing will include reorientation to the anterior–posterior commissure, normalization to template space, segmentation into tissue types (grey matter (GM), white matter (WM) and cerebrospinal fluid (CSF)), calculation of total intracranial volume and smoothing. Because the participant group is pediatric, as recommended in the CAT12 manual (86), the use of custom tissue probability maps and a custom DARTEL template will be explored. For statistical analyses, we will use ANCOVA with post-intervention grey and white matter volume as dependent variables and intervention, total intracranial volume, age, and IQ as covariates. Participants’ relative GM and WM volume changes will be assessed at a threshold of *p* < 0.05 and will be corrected for multiple comparisons (family-wise error).

Analysis software will be R (77), SPM12 on MATLAB (87) for standard preprocessing, the CAT12 toolbox and DARTEL tools in SPM12 for analysis of VBM, and CONN for denoising and RSFC analysis.

#### 2.7.4. Gut-microbiome data analysis

For the gut-microbiome analysis, the microbial community composition in collected stool samples will be determined by ribosomal small subunit (SSU rRNA/ 16S rRNA) gene amplicon sequencing (76). Genetic sequencing work and data preprocessing will be carried out by the Joint Microbiome Facility (University of Vienna and Medical University of Vienna). In short, DNA extraction with the QIAamp DNA Stool Mini Kit will be automated on a QiaCube. For microbial community profiling, the 16S rRNA genes will be amplified by polymerase chain reaction (PCR) applying primers which cover most bacterial and archaeal clades (515F, 806R) (88). After PCR amplification of the marker gene region, the amplicons will be barcoded, multiplexed and sequenced on the Illumina MiSeq platform at the Joint Microbiome Facility (76). Negative controls will be performed during sampling, DNA extraction, and barcoding. The obtained amplicon sequence data will then be quality-filtered and demultiplexed, followed by amplicon sequencing variant (ASV) inference with DADA2 (89), enabling analysis at the highest possible taxonomic resolution. Resulting ASV sequences will be taxonomically classified using SINA (90) with the newest release of the SILVA SSU rRNA database (91). If necessary, contaminants will be removed *in silico* using the decontam software package (92).

Abundance measurements (counts) of ASVs, as well as ASV sum counts at higher taxonomic levels will be statistically evaluated, to test for significant differences in microbial community composition in samples retrieved before and after intervention. Detection of significantly more abundant amplicon sequence variants after intervention compared to before intervention will be performed, and adjusted *p*-values will be calculated using the Benjamini-Hochberg method. Differences supported with *p*-values <0.05 will be considered significant. Statistical analysis will be performed with the metagenomeSeq software, which has been proven optimal for amplicon datasets (93). MetagenomeSeq normalizes the abundance data to address varying depths of sequencing coverage across samples, and then a zero-inflated log-normal mixture model is applied to calculate the fold changes between the case and control group for each taxonomic level (94).

### 2.8. Data collection and management

All collected data will be stored on a password-restricted server. Survey data will be collected and managed using REDCap electronic capture tools hosted at NORCE (95). Data will be entered directly by parents or teachers on the REDCap server or independently entered twice by researchers if collected on paper. Data storage and analysis for both sites will be conducted on a dedicated server at the University of Bergen, protected by multi factor authentication to ensure a homogenous analysis pipeline and adequate safety of participant data.

De-identified clinical and neuroimaging data will be made accessible for re-use by other researchers via platforms such as ENIGMA. De-identified clinical data will also be stored in a publicly available repository (Open Science Foundation^1^).

#### 2.8.1. Biological samples

Upon receival of the stool samples at the MRI scanner, researchers will disinfect the containers and transport the samples to a freezer where they will be stored at a temperature between −20 and−30 degrees Celsius. After data collection has finished at both sites, the Norwegian samples will be transferred from Bergen to Vienna on dry ice for analysis.

The hair samples will be transported from the room of the study visit to a storage room where they will be kept at room temperature in a dry and dark locked cabinet. After data collection has finished at both sites, the Norwegian samples will be transferred from Bergen to Vienna for analysis.

## 3. Discussion

The Music for Autism (M4A) trial is designed to replicate and expand upon the only previous RCT on neurobehavioral outcomes of music therapy for school-aged children with autism. Anticipated risks, strengths and limitations of the study design, and the potential impact of findings are discussed below.

### 3.1. Risk mitigation

Slow recruitment is the biggest risk for the trial. The teams in Bergen and Vienna can draw on a history of successful recruitment for ASD studies (30) that support the feasibility of recruitment for M4A. We anticipate slower recruitment in Bergen relative to Vienna given that Bergen is a smaller city with a smaller population. Thus, recruitment can be extended from Bergen to other cities if needed throughout the trial. Furthermore, participation in the trial requires the ability to lay still in the scanner, which can be difficult for many children who would otherwise be eligible. To increase the likelihood of participation, we will offer tools and preparation for eligible children and families prior to the baseline scan.

An additional set of challenges is associated with the trial being a multinational replication study. These include language barriers, the availability of assessments in each language, but also variations in scoring procedures and scales requiring adaptations for consistency. The CCC-2 in Vienna; PPVT-4 in Norway; CASP in Vienna; and FQoL in both countries, will be translated in-house. The measures will be translated and back translated by independent researchers to ensure the accuracy of the content.

Adequate timing of interventions and follow-ups in trials such as the M4A provides logistical challenges as it requires the collaborative and timely effort of many people involved – therapists, parents, teachers, children, assessors, radiologists, and researchers. To ensure timely completion of assessments, planning and follow-up procedures will be implemented across sites.

### 3.2. Strengths and limitations of the study design

An important strength of M4A is its randomized design, which allows for causal conclusions and is considered the gold standard design for evaluating intervention effects. The crossover design implemented in M4A offers the advantage of requiring fewer participants to attain sufficient statistical power while allowing for the examination of individual preferences based on their experience with both interventions (79). However, it should be noted that children are not asked directly about their preferences, but rather parents are asked to report their child’s preferences. Moreover, crossover designs require certain assumptions that can be challenging to assess, including the chronicity and stability of the condition, reversibility of outcomes, and the duration of intervention effects. Although ASD is a lifelong condition, associated behaviors may not remain constant over time, particularly in children. Furthermore, the interventions are hoped to lead to lasting learning effects, which can potentially impact the comparability of the second baseline to the initial one. Moreover, the relative benefits of each intervention may depend on the order in which they are received.

However, we aim to mitigate these issues by implementing a relatively long wash-out period that matches the intervention duration. A practical consideration is the extended timeframe required for each participant, as families might be more motivated to participate in a study where they have the opportunity to receive both interventions compared to a parallel study where their preferred intervention may not be available.

An additional limitation of the study design is that the duration of the interventions might not be sufficient to observe changes. However, given that the study by Sharda et al., which the M4A trial aims to replicate, found effects on functional brain connectivity, family quality of life, and social communication, following 8–12 weeks of music intervention, we expect to replicate these findings. Additionally, a large multinational RCT on music therapy for children with autism, the TIME-A (30), evaluated dosage effects and found no effect of increased duration of interventions on the outcome measures. However, it is important for further studies to assess if a dosage effect is observed, particularly when integrating outcomes from multiple domains, such as functional connectivity and other biological outcomes.

Finally, it is important to note that the power calculations for the M4A study were conducted for the primary outcomes, so analyses involving secondary outcomes, relationships between biomarkers and psychometric outcomes, and clinically defined subgroups will be considered exploratory.

### 3.3. Potential impact

By conducting a replication study in a different cultural context, employing a larger sample size, and incorporating additional measures, the M4A trial may reinforce the initial findings of improved social communication and resting state functional connectivity among a cohort of school-aged children with autism following a music therapy (MT) intervention (29). Additionally, the study has the potential to advance our understanding of the biological mechanisms underlying MT and its associations with psychometric outcomes.

MT is considered a low-risk intervention for children with autism, as it has minimal side effects (11, 30) and is often more accessible compared to invasive approaches like medication or costly interventions such as early intensive behavioral intervention. Despite the positive outcomes indicated by recent systematic reviews on MT for individuals with autism, there remains a need for further studies to explore the impact of MT on functional brain connectivity. Currently, the existing empirical studies focusing on MT’s effects on brain connectivity in children with autism are limited to the trial conducted by Sharda et al., which the M4A study aims to replicate. By specifically isolating the effects of MT, M4A’s findings hold the potential to enhance our understanding of how MT influences functional brain connectivity as well as other biological outcomes among school-aged children with autism.

Considering the inclusive nature of M4A, which aims to recruit children with varying levels of functioning, the study results have the potential to offer valuable insights into the effects of MT on different subgroups within the autism population. This, in turn, could contribute to a more refined selection of suitable cases. Understanding the complex relationships between clinically or neuroscientifically defined subgroups and treatment outcomes requires additional analyses. Advanced techniques such as machine learning can be applied to extract meaningful patterns from the comprehensive dataset. Moreover, data-driven methods, including resting-state fMRI analysis techniques like graph theory and dynamic functional connectivity, can offer alternative ways to process and interpret the data.

## 4. Ethics and dissemination

### 4.1. Ethics

The study was approved by the Norwegian Regional Committees for Medical and Health Research Ethics (REK Sør-Øst D, 07 May 2021, reference 246,145) and the Ethics Committee of the University of Vienna (21 April 2021, reference 00634) before the start of enrollment. Upon recruitment into the study, parents/caregivers of eligible participants will provide informed consent. Children will assent orally based on oral information and an invitation letter describing the study in simple terms. Terminating participation in the study is possible at any time during the process.

### 4.2. Community involvement

User representatives (individuals with autism and their family members) will be involved in all phases of the study. They gave advice on the relevance of intervention elements, primary and secondary outcomes. They will continue to give advice to the project team, assist with recruitment and dissemination of the results.

### 4.3. Dissemination

Our dissemination plan includes various channels to effectively reach both academic and non-academic audiences. We aim to disseminate our study results by publishing in high-quality scientific journals specializing in autism, child psychiatry, music therapy, neuroscience, and related fields. Active participation in national and international conferences in relevant fields will also be an integral part of our dissemination strategy.

We recognize the importance of engaging and informing key stakeholders, including parents, caregivers, and professionals involved in supporting children with autism. To raise public awareness about our research we will engage with the wider community through public talks, media interviews, and collaborations with local organizations. By disseminating our findings to the public, we aim to foster a greater understanding of the potential benefits of music therapy for children with autism.

Digital platforms will play a key role in effectively disseminating our research outcomes. We will maintain a dedicated project website, providing regular project updates, and access to publications. Additionally, we will use various social media channels to engage with a wider audience by sharing noteworthy study highlights and to address any inquiries.

## 5. Trial status

Recruiting. Inclusion of participants started in September 2021. By June 2023, 53 participants have been included and randomized into the study.

## Ethics statement

The study was reviewed and approved by the Norwegian Regional Committees for Medical and Health Research Ethics (REK Sør-Øst D, 07 May 2021, reference 246145) and the Ethics Committee of the University of Vienna (21 April 2021, reference 00634) before the start of enrollment. Upon recruitment into the study, parents/caregivers of eligible participants will provide written informed consent; children will assent orally based on oral information and an invitation letter describing the study in simple terms. Terminating participation in the study is possible at any time during the process.

## Author contributions

MR: Conceptualization, Investigation, Project administration, Writing – original draft, Writing – review & editing. AGr: Conceptualization, Investigation, Project administration, Writing – review & editing. AGu: Conceptualization, Software, Writing – review & editing. AK: Writing – review & editing. NM: Conceptualization, Methodology, Writing – review & editing. UN: Conceptualization, Methodology, Writing – review & editing. KK: Methodology, Software, Writing – review & editing. M-BP: Conceptualization, Project administration, Supervision, Writing – review & editing. MS-G: Project administration, Writing – review & editing. BT: Data curation, Methodology, Supervision, Investigation, Writing – review & editing. IW: Conceptualization, Methodology, Writing – review & editing. CG: Conceptualization, Funding acquisition, Investigation, Methodology, Project administration, Supervision, Writing – original draft, Writing – review & editing. GS: Conceptualization, Funding acquisition, Methodology, Project administration, Writing – review & editing. KS: Conceptualization, Funding acquisition, Methodology, Project administration, Supervision, Writing – review & editing.

## Footnotes

^1^https://osf.io/
