# Supplementary material for: Music for autism: a protocol for an international randomized crossover trial on music therapy for children with autism
Source: Front Psychiatry. 2023 Oct 2;14:1256771. doi: 10.3389/fpsyt.2023.1256771 (PMC10598663; doi:10.3389/fpsyt.2023.1256771)
Supplement: Supplementary file 1 [file Data_Sheet_1.docx]

Supplementary Material

# Supplementary Figures and Tables

## Supplementary Figures

**Supplementary Figure 1. Visual schedule used to facilitate scanning used in Bergen (example)**

A trip to the MR

| 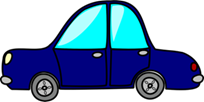 | Drive car to Haukeland |
| --- | --- |
| 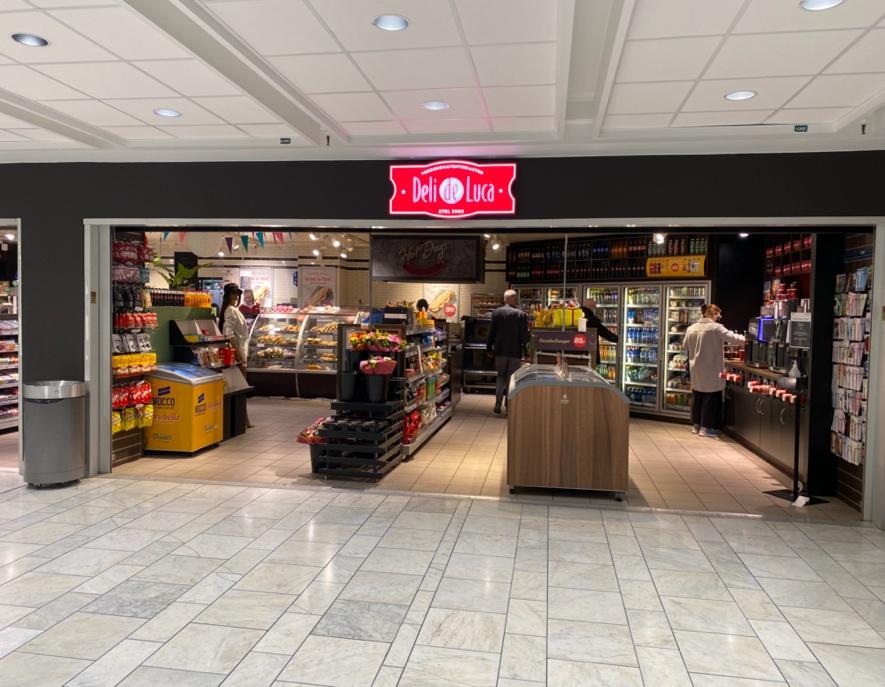 | Go to Deli de Luca and wait for Marianna |
| 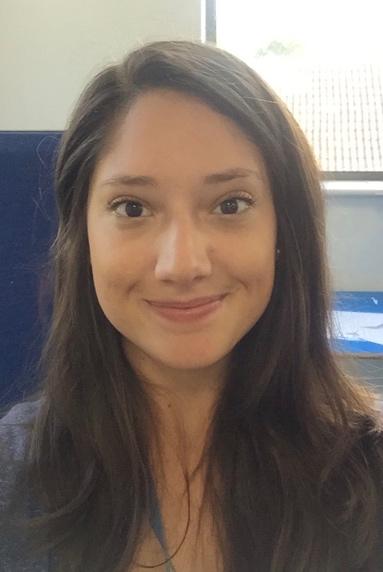 | Meet Marianna |
| 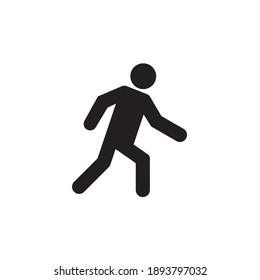 | Walk together to the MR U3 room |
| 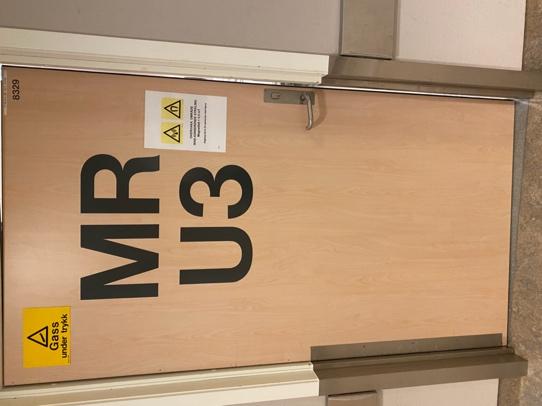 | Go in the door MR U3 |
| 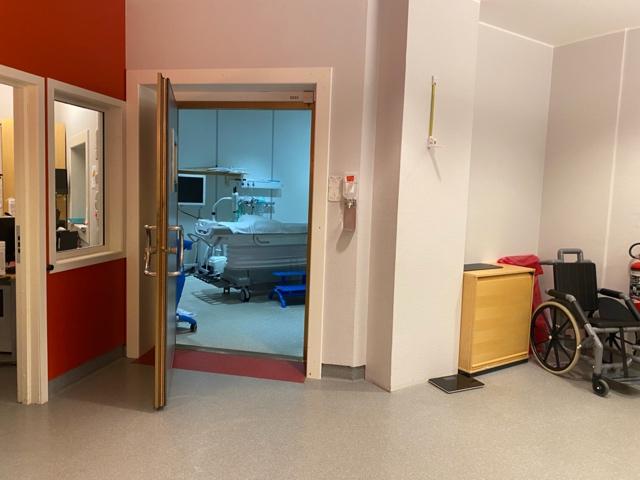 | Go in the room |
| 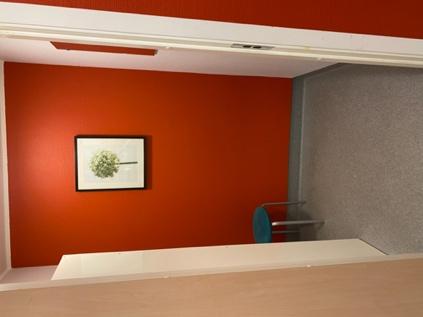 | Go in the closet and take off jacket |
| 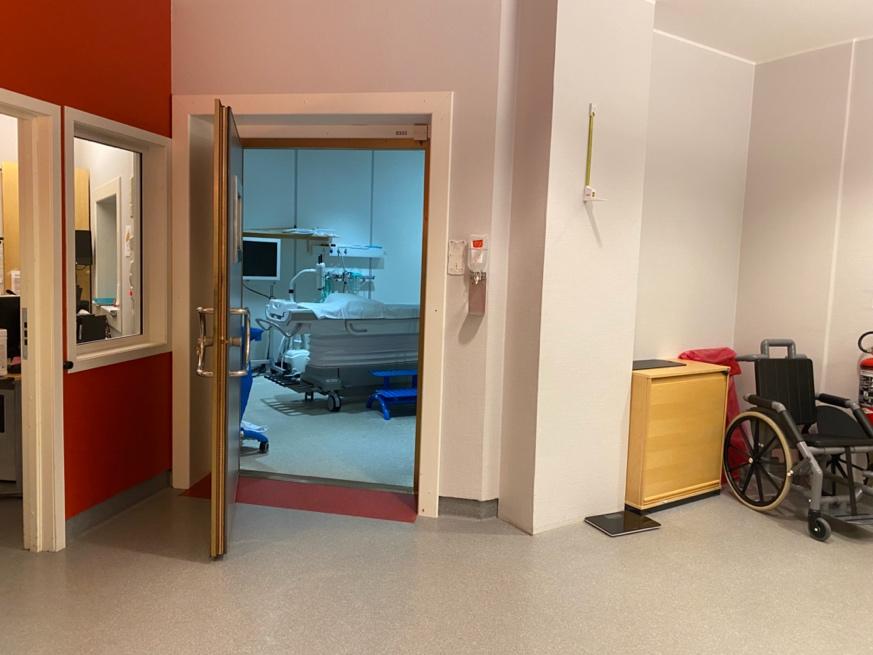 | Go in the door to the MR room |
| 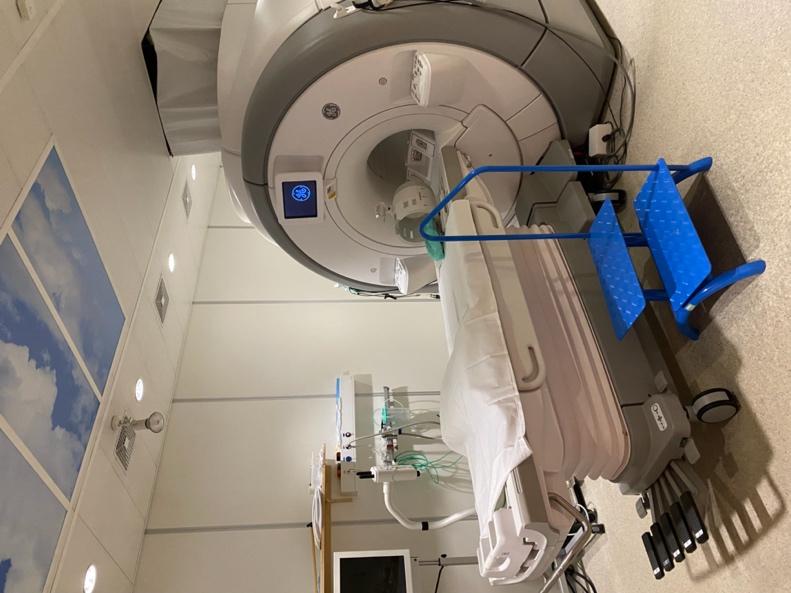 | Lay on bench |
| 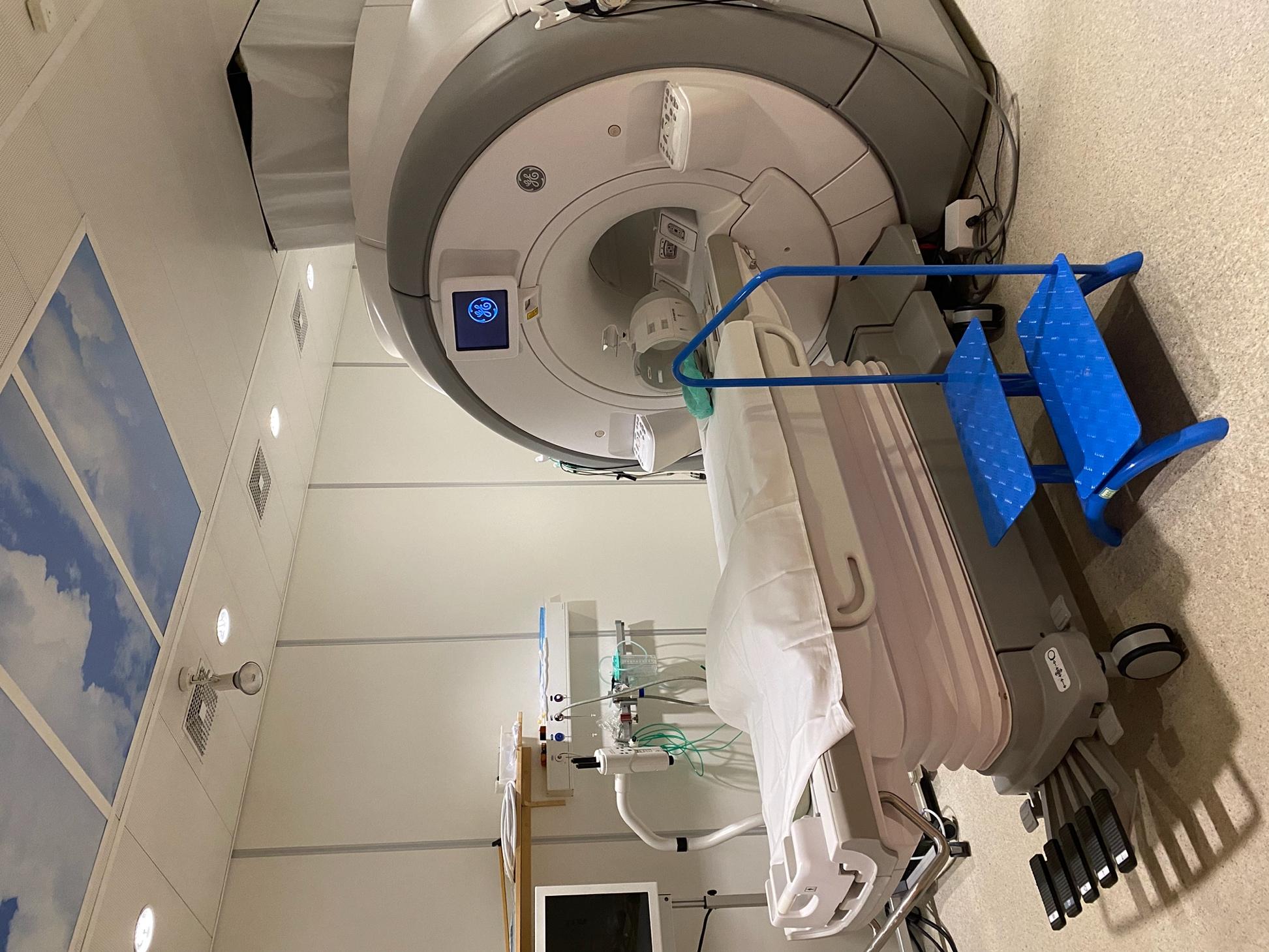 | Put head on the pillow in the machine |
| 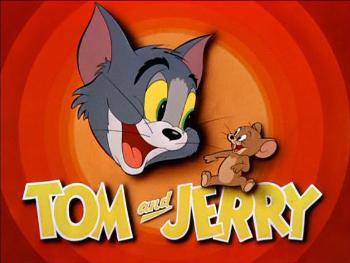 | Watch Tom and Jerry |
| 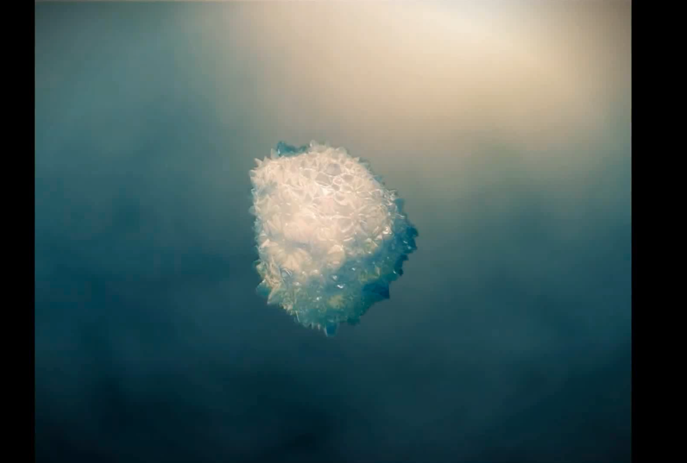 | Watch a film and listen to music |
| 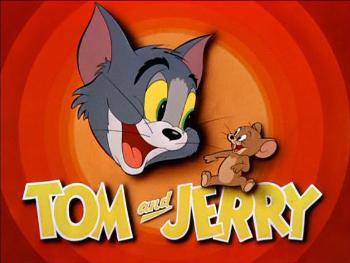 | Watch Tom and Jerry one more time |
| 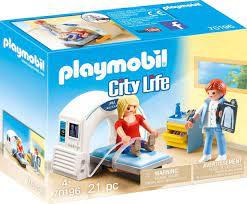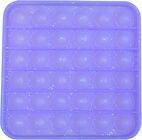 | Receive a prize |
| 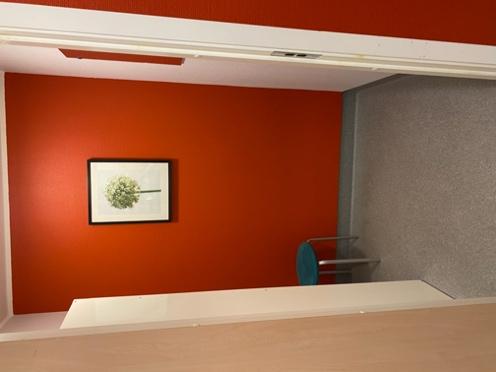 | Put clothes on |
| 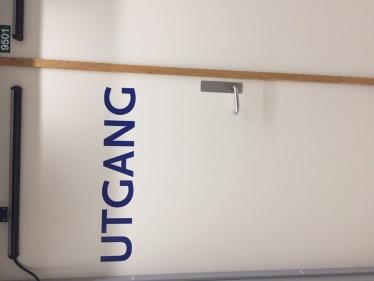 | Go out the door that says Exit |
| 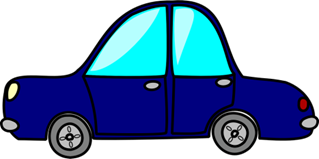 | Go in the car |

*Note*. Similar, individually adapted visual schedules were used for different children.

## Supplementary Tables

**Supplementary Table 1. List of changes to the protocol**

| **Date** | **Change** |
| --- | --- |
| 31 August 2020 (V1) | Original proposal submitted to Kavli Trust |
| 11 February 2021 (V2) | New project start date |
| 24 August 2021 (V3) | Microbiome data added |
| 04 November 2021 (V4) | Prediction tasks added; “NM” changed to “PT” in text and flow-chart |
| 14 January 2023 (V5) | Scoring specifications added and updated; text revised; outdated figures removed; SPIRIT schedule figure added; implementation results added; extended recruitment period |

**Supplementary Table 2. Scenarios reaching 80% power in M4A**

| Scenario | *d* | *r* | *n* | Power |
| --- | --- | --- | --- | --- |
| A | 0.34 | 0.50 | 70 | 80% |
| B | 0.30 | 0.60 | 70 | 80% |
| C | 0.37 | 0.40 | 70 | 80% |
| D | 0.34 | 0.60 | 56 | 80% |
| E | 0.34 | 0.40 | 83 | 80% |
| F | 0.34 | -- | 274 | 80% |

*Note*. Showing alternative scenarios that lead to 80% power. Scenarios A to E show varying assumptions for effect size d and correlation r with the present crossover design. Scenario F shows the much larger sample size that would be needed in a parallel design to reach the same power.
